# A Logarithmic Formulation for Anisotropic Behavior Characterization of Bovine Cortical Bone Tissue in Long Bones Undergoing Uniaxial Compression at Different Speeds

**DOI:** 10.3390/ma14175045

**Published:** 2021-09-03

**Authors:** Abdallah Shokry, Hasan Mulki, Ghais Kharmanda

**Affiliations:** 1 Department of Mechanical Engineering, Faculty of Engineering, Fayoum University, Fayoum 63514, Egypt; abdallah.shokry@fayoum.edu.eg; 2 Smart Engineering Systems Research Center (SESC), Nile University, Shaikh Zayed City 12588, Egypt; 3 College of Engineering and Technology, American University of Middle East, Egaila 15453, Kuwait; 4 Mechanics Laboratory of Normandy, INSA Rouen, 76800 St Etienne du Rouvray, France; mgk@scs-net.org

**Keywords:** bone material characterization, high strain rates, transversal isotropy, anisotropic behavior, long bones

## Abstract

The mechanical properties of bone tissues change significantly within the bone body, since it is considered as a heterogeneous material. The characterization of bone mechanical properties is necessary for many studies, such as in prosthesis design. An experimental uniaxial compression study is carried out in this work on bovine cortical bone tissue in long bones (femur and tibia) at several speeds to characterize its anisotropic behavior. Several samples from different regions are taken, and the result selection is carried out considering the worst situations and failure modes. When considering different displacement rates (from 0.5 to 5 mm/min), three findings are reported: The first finding is that the behavior of bone tissues in radial and tangential directions are almost similar, which allows us to consider the transversal isotropic behavior under static loads as well as under dynamic loads. The second finding is that the failure stress values of the longitudinal direction is much higher than those of the radial and tangential directions at low displacement rates, while there is no big difference at the high displacement rates. The third finding is a new mathematical model that relates the dynamic failure stress with the static one, considering the displacement rates. This model is validated by experimental results. The model can be effectively used in reliability and optimization analysis in prosthesis design, such as hip prosthesis.

## 1. Introduction

The mechanical characteristics of bone rely on composition and structure. Nevertheless, the composition of living tissues varies. It is continually changing due to the mechanical forces, ageing, illness, nutrition and other considerations [1]. In general, bone mechanical properties are estimated at relatively low displacement rates. Conversely, real life bone often fails because of accidental impacts from instances of locomotion, athletics, sports or coach accidents. As a result, when a shock is applied to cortical bone, it tears, damages and fails. Consequently, the quasi-static loading is insufficient for utilization in accidentology and traumatology. This way, when dealing with prosthesis designed especially for young people, the mechanical properties of bone under static loading may lead to unacceptable designs. Therefore, there is a strong need to model these properties under quasi-static or dynamic loading, especially long bone such as femur and tibia. Few works in the literature have reported the consideration of high displacement (strain) rates, with more of a focus on the axial direction only [2]. In this work, dynamic loads are considered to define the relationship between the failure stress values in dynamics and the corresponding values in statics. In Kharmanda [3], an optimized formulation that relates the yield stress with the modulus of elasticity has been elaborated for isotropic bone material. On the other hand, in Kharmanda et al. [4], it was extended to transversal isotropic bone material where the radial and tangential directions are considered almost the same. In order to take the quasi-static and/or dynamic loading into account, a new formulation is needed to relate the static failure stress with the dynamic one.

In fact, experimental studies on fresh bones are restricted by the difficulties of collecting specimens. In addition, the sophistication of testing procedures makes the prediction of the mechanical behavior of bone a difficult task. However, the integration of dynamic studies in numerical simulation procedures is strongly needed, and leads to much more realistic results. When considering a long bone as a cylinder, three directions should be taken into account: radial, axial and tangential. In this work, a dynamic loading is considered to identify the bone behavior. A compression test is performed on several samples to identify the cortical bone behavior in the three directions: radial, axial and tangential. The different studies consider that the most important loading on the long bone is the axial loading, while when using these prostheses in young people, this consideration limits the movements of the prostheses. Therefore, there is a need to take into account the loading in the other directions. In our previous studies [3,4], we focused on prosthesis design, considering only static loads. In order to simulate the gait cycle, a dynamic analysis should be involved in the design process, in which data and formulations are necessary to characterize the material properties in dynamics to obtain realistic results. One of the most important applications of this work is the orthopedic prosthesis design, where the dynamic loading needs to be considered during the design process to avoid or to minimize the risk of the implant loosening and bone and/or implant fracture. In this way we increase the prosthesis reliability and safety levels, and decrease the probability of revision surgeries. Another important finding is the demonstration of the transversal isotropy property in dynamic loading cases as well as in static ones.

## 2. Literature Review

In the literature, several studies (bovine and human bone studies) have been performed to identify the mechanical properties under static and dynamic loads at macro- and microstructure levels.

In bovine bone studies, Ferreira et al. [5] studied the effect of various levels of high strain rates on the mechanical properties of fresh cortical bone. They described its characterization under different loading conditions. Their results show that the elastic modulus decreases, and the ultimate stress increases as the strain rate increases in both longitudinal and transversal directions. The mechanical properties as well as the microstructure of young and adult bovine bone were studied by Manilay et al. [6]. The characterization was performed in longitudinal and transversal directions using optical microscopy, and the mechanical properties were investigated using compression testing for untreated, deproteinized and demineralized cases. Their results show that the mature bone has higher values of compressive strength and lower values of toughness compared to a young bone in an untreated case. Thus, in a deproteinized case, mature bone has shown to be stronger than young bone in longitudinal and transversal directions. On the contrary, the young bone is found to be stronger than the mature bone in demineralized cases in longitudinal and transversal directions. Li et al. [7] studied the mechanical properties of cortical bovine bone using tension and compression tests and various loading modes. Their results confirmed that bone withstands compressive strength rather than tensile strength. Nobakhti et al. [8] investigated the effect of elastic modulus on bovine femur, in the samples that were taken from proximal and metaphysics, and tested them using three-point bending. They found that the elastic modulus changes along the femur, which is comparable as described by Wolff’s law.

In human bone studies, Hansen et al. [9] investigated the impact of strain rate on both tensile and compressive characteristics of human bone. They found that a quite simple linear relationship might be found between yield properties and strain rate, however such relationships between post yield properties and strain rate were not found. Consequently, the strain rate has a larger and greater effect on post yield deformation, rather than its effect on initiation of yielding. Boskey and Coleman [10] studied the influence of aging on the mechanical and geometrical properties of bones. The change in the function throughout life could be improved or deteriorated. Donnelly [11] reviewed various methods of assessing bone quality (geometric and material factors). Different methods for assessing mechanical properties, from whole bone to the nanoindentation testing technique, were compared. She concluded that no single method can completely describe the bone quality. Thus, a combination of noninvasive imaging techniques with ex vivo mechanical and compositional techniques were recommended. Cole and van der Meulen [12] reviewed the whole-bone mechanics and behavior, considering the intrinsic and extrinsic characteristics. They concluded that the laboratory tests of the whole bone strength are the only measure of in vivo fracture prediction, although the combined imaging models may be used for strength prediction. Zimmermann and Ritchie [13] and Zimmermann et al. [14] studied the bone structure and the influence of aging, disease and treatment on the mechanical properties of human bone (fracture). It has been found that the reduced mechanical integrity is generated from alterations to the hierarchical structure. Lughmani [15] concentrated on bone drilling using experimental and numerical approaches. The efficacy of using bone drilling data was considered as an indicator to estimate bone quality. Brown et al. [16] and Gauthier et al. [17] studied the influence of loading conditions (quasi-static loading) on human bone mechanical properties. As a result, the bone mechanical properties depend on the location and strain rate.

## 3. Materials and Methods

**Figure 1:**
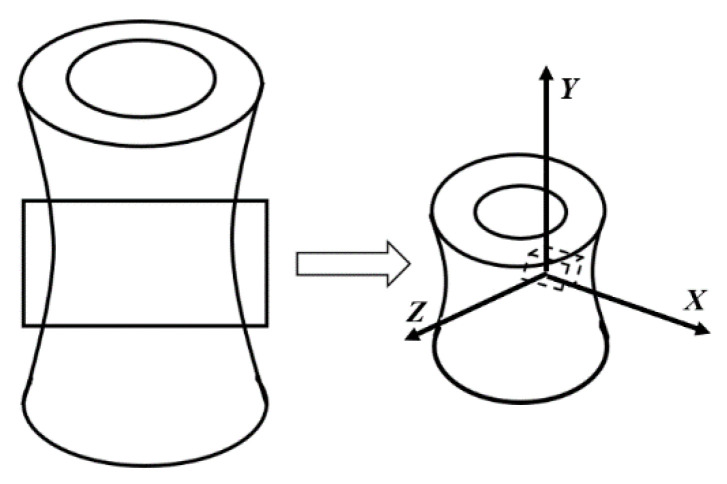
Radial, axial and tangential directions in the studied long bone.

Samples of cortical bovine bone were taken from the midsection of long bones of the Holstein cow. The long bone was obtained from a local butcher shop in Lund, Sweden, in which fresh bone can be obtained. The samples were cut to be in cubic shapes using a cutting machine (Struers Accutom-5) that is located in the Solid Mechanics Laboratory at Lund University, and then the marrow was removed from each sample. In order to study the anisotropic properties of the bone, the x-axis and the y-axis were given to the radial and longitudinal directions, respectively, while the z-axis was given to the tangential direction (see Figure 1) in the cubic samples (more than 100 samples).

**Figure 2:**
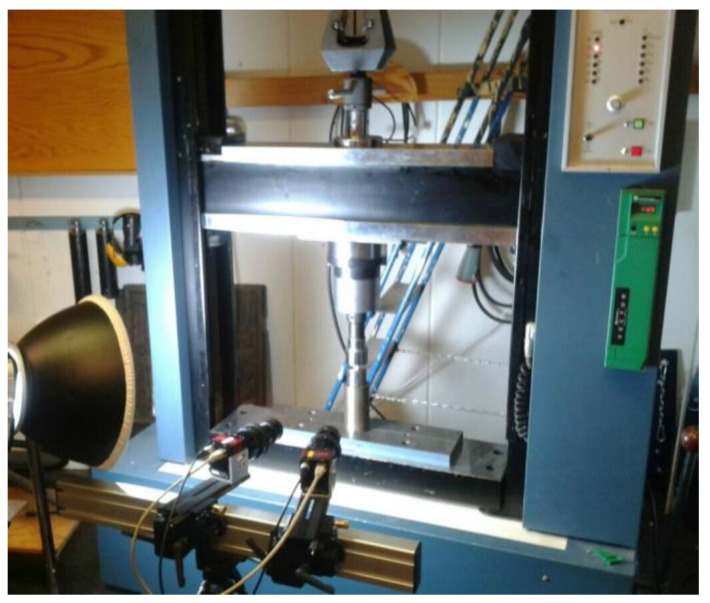
Compression machine at the Solid Mechanics Laboratory.

The cubic bone samples were prepared with a mean value of 10 mm for the x, y and z with standard deviations of ±2 mm for x, y and z, respectively. The samples were stored in a freezer at −2 °C. On the test day, the samples were placed at room temperature for one hour. For the purpose of studying the behavior of the bone at different loading rates, a number of compression tests at different speeds were made using a universal testing machine (MTS 810 servohydraulic machine) (see Figure 2), which was located in the Solid Mechanics Laboratory at Lund University, and has a capacity of 100 kN with the following displacement rates 0.5, 1, 2, 5 and 10 mm/min. The fifth displacement rate (10 mm/min) led to unstable results. Therefore, only the first four speeds were implemented in this research.

The density had a large influence on the failure process, so samples with almost the same density were considered to fit the experimental curves. Samples were then cut into a cubic form with length (a≈10 mm), with a numerically controlled machine tool. In this test, the relationship between the compression strength and loading speed was modeled. The tests were performed for several samples. For the mathematical model elaboration, the density range tolerance was small (±0.005 g/cm^3^). However, it was much more difficult to select several samples with the same density considering the same range, to carry out the validation for all directions and several speeds. Therefore, an increase in the range variation was considered. This range tolerance was: ±0.05 g/cm^3^ since it seems to be very difficult to get same densities for different speeds.

## 4. Results

### 4.1. Experimental Results

More than one hundred samples were subjected to a compression test in three directions: x, y and z (radial, axial and tangential directions) and at four different speeds: $v_{1}=$ 0.5, $v_{2}=$ 1, $v_{3}=$ 2 and $v_{4}=$ 5 mm/min. The samples were cut from different regions of the femur and tibia bones. At the first speed, almost 20 (different density values) for each direction, and almost 36 samples for the other speeds were tested. There were also some additional samples which were immediately broken and led to unrealistic results. These samples might have had problems during the cutting process from the long bone, such as obtaining samples with non-flat surfaces, and the presence of significant pores inside samples that was discovered after the test in the damaged samples. These samples were eliminated from this study. In addition, since the main objective of design prosthesis (such as in hip replacement) is to obtain reliable structures, it is better to take the worst scenario into account, and here the worst scenario is a failure of the bone at lower stress values.

According to these experiments, it was found that when increasing the displacement rate values, the failure stress values decreased in certain samples, while they increased in other samples. To get a reliable model as mentioned above, the samples that led to a decrease in the failure stress values are only considered. Another consideration is that the selected samples in the same direction must have had the same density. Moreover, to compare between the three directions, the samples subjected to the same displacement rates were selected. Furthermore, knowing if the transversal isotropy can be applied in dynamics as well as in statics is an important issue to be studied.

For the radial direction (x), the interval of density was: $\rho =\left[1.63-2.1\right]{\;g/\mathrm{cm}}^{3}$. Three samples with the same density ($\rho =1{.87\;g/\mathrm{cm}}^{3}$) at three displacement rate values ($v_{1}=$ 0.5, $v_{2}=$ 1 and $v_{4}=$ 5 mm/min) were selected. Furthermore, for the axial direction (y), the interval of density was: $\rho =\left[1.46-2\right]{\;g/\mathrm{cm}}^{3}$. Three samples with the same density ($\rho =1{.70\;g/\mathrm{cm}}^{3}$) at three displacement rate values ($v_{1}=$ 0.5, $v_{2}=$ 1 and $v_{4}=$ 5 mm/min) were selected. Thus, for the tangential direction (z), the interval of density was: $\rho =\left[1.68-2.48\right]{\;g/\mathrm{cm}}^{3}$. Three samples with the same density ($\rho =1{.69\;g/\mathrm{cm}}^{3}$) at three displacement rate values ($v_{1}=$ 0.5, $v_{2}=$ 1 and $v_{4}=$ 5 mm/min) were selected. The experimental values of the failure stresses at three displacement rate values for the three directions are presented in Table 1.

### 4.2. Proposed Formulation

**Figure 3:**
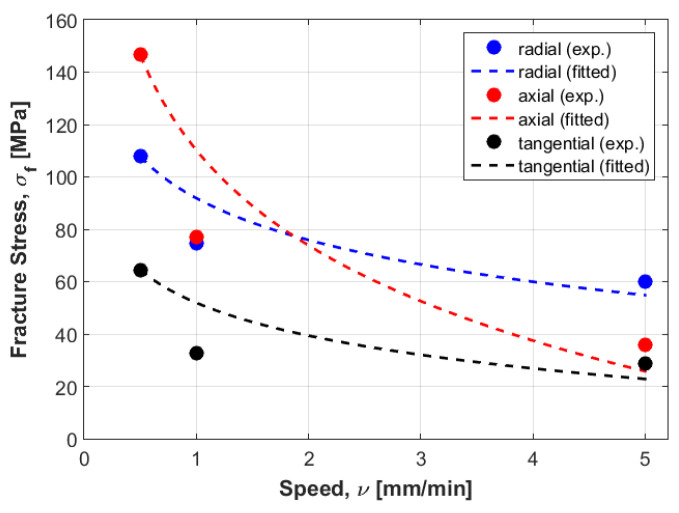
Experimental fracture stress in dots and curve fitting of the three directions at the three displacement rate values.

To mathematically model these results, logarithmic formulation that relates dynamic fracture stress with static fracture stress was suggested for the three directions: radial, axial and tangential. Figure 3 represents the experimental fracture stress in dots and curve fitting of the three directions at the three displacement rate values.

A mathematical model that correlates static fracture stress, dynamic speed and static speed to dynamic fracture stress can be represented as follows:

(1) $$\sigma_{d}=\sigma_{s}\left(1+C_{i}\ln\left(\frac{v_{d}}{v_{s}}\right)\right)$$

where $\sigma_{d}$ represents dynamic fracture stress, $\sigma_{s}$ represents static fracture stress, $v_{d}$ and $v_{s}$ represent dynamic speed and static speed, respectively. $C_{i}$ is a constant and *i* stands for radial, axial and tangential directions. In order to determine the constant $C_{i}$, Equation (1) can be rearranged as follows:

(2) $$\frac{\sigma_{d}}{\sigma_{s}}=1+C_{i}\ln\left(\frac{v_{d}}{v_{s}}\right)$$

**Figure 4:**
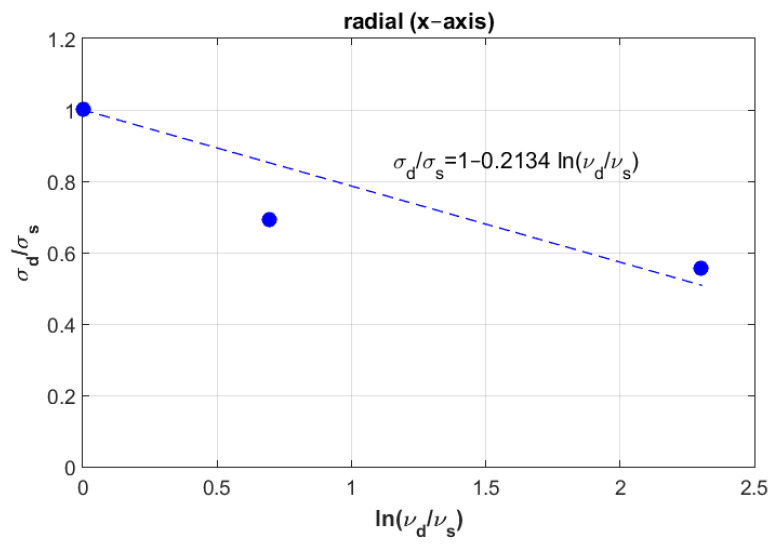
Determination of constant $C_{i}$ in the radial direction (X).

**Figure 5:**
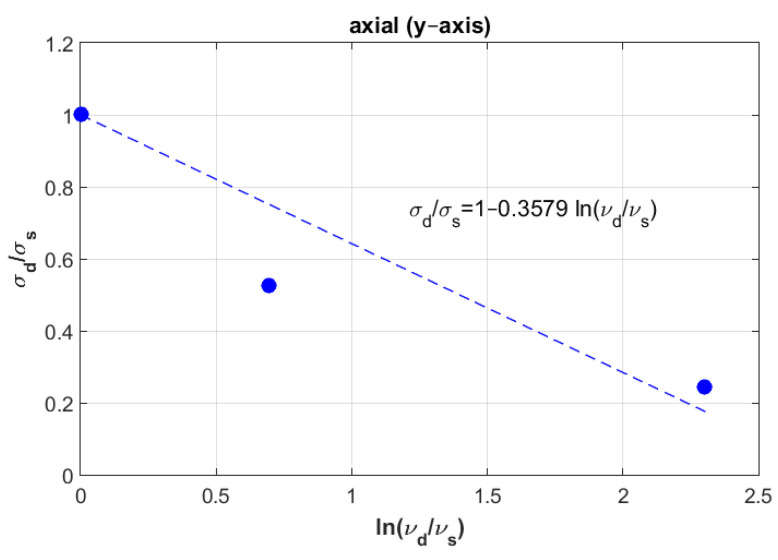
Determination of constant $C_{i}$ in the axial direction (Y).

**Figure 6:**
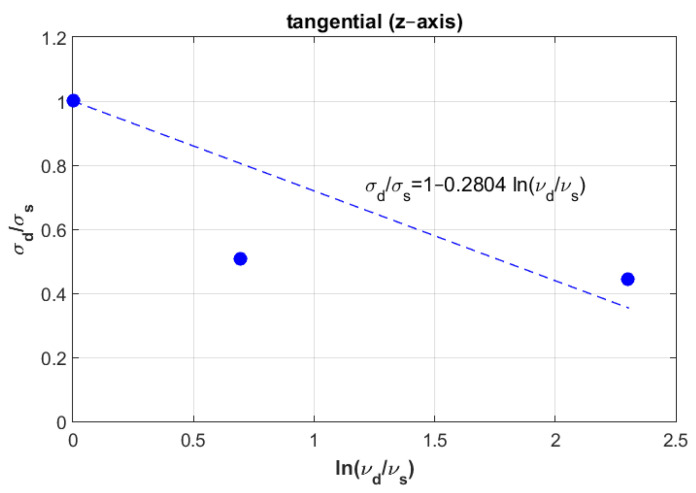
Determination of constant $C_{i}$ in the tangential direction (Z).

Which can be graphically plotted as a linear equation with an intercept equal to one and a slope equal to $C_{i}$. Figure 4, Figure 5 and Figure 6 show the ratios $v_{d}/v_{s}$ versus $\sigma_{d}/\sigma_{s}$ for the three directions radial, axial and tangential, respectively.

Table 2 shows the resulting values of the constant $C_{i}$ for the three directions radial, axial and tangential.

A comparison between the experimental fracture stresses and the corresponding predicted stresses using the suggested mathematical model is presented in Table 1.

### 4.3. Validation of Experimental Results Using Finite Element Method (FEM)

In order to validate the different results, a large number of experiments were required since the study was carried out in three directions and at different speeds. Hence, numerical simulations were implemented to analyze the effect of data spread, considering the limited number of experiments that were conducted in this work. A refined FEM will definitely help to make an effective analysis, by reducing the amount of needed measured data. The finite element (FE) models require some additional data. For example, the modulus of elasticity was needed in all directions (*x*, *y*, *z*) at the same density ($\rho =1{.8\;g/\mathrm{cm}}^{3}$). The experimental stress for radial, axial and tangential corresponds to three samples, with one sample for each direction. However, due to availability of samples with the same density, the Young’s modulus was calculated as a mean value of two samples in each direction at the same density. The mean resulting Young’s modulus values in the three directions are: *E_x_* = 1969.83 MPa, *E_y_* = 4711.88 MPa and *Ez* = 1899.66 MPa.

**Figure 7:**
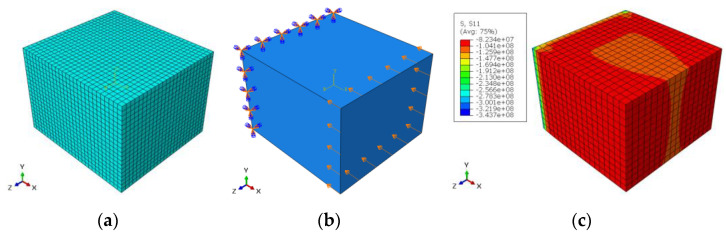
(**a**) FE model, (**b**) boundary conditions and (**c**) stress distribution for radial direction.

For radial direction, the dimensions of the selected sample in the three directions were: 10.88 × 7.85 × 10.07 mm, when the experimental fracture stress was 91.71 MPa (see Table 3). The mesh model contained 13,500 elements when the element type was C3D20R (20-node quadratic brick). The number of nodes was: 59,290. The convergence study for element numbers gives less than 1% with element size = 0.0004 m. For the boundary conditions, the displacement in x-direction was: 0.000507 m in compression and the left surface in y–z plane was prevented from movement and rotation in x, y and z directions. Figure 7a–c show the FE model, the boundary conditions and the stress distribution for radial direction, respectively.

**Figure 8:**
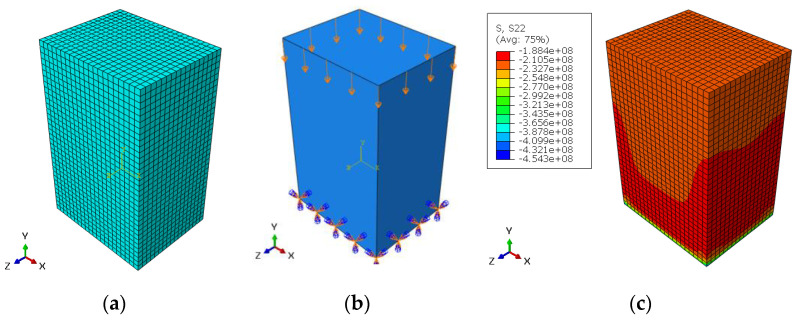
(**a**) FE model, (**b**) boundary conditions and (**c**) stress distribution for axial direction.

For the axial direction, the dimensions of the selected sample in the three directions were: 8.4 × 13.48 × 6.73 mm when the experimental fracture stress was 160.97 MPa (see Table 3). The mesh model contained 12,138 elements when the element type was C3D20R (20-node quadratic brick). The number of nodes was: 53,644. The convergence study for element numbers gives less than 1% with element size = 0.0004 m. For the boundary conditions, the displacement in y-direction was: 0.0004605 m in compression and the lower surface in x–z plane was prevented from movement and rotation in x, y and z directions. Figure 8a–c show the FE model, the boundary conditions, and the stress distribution for axial direction, respectively.

**Figure 9:**
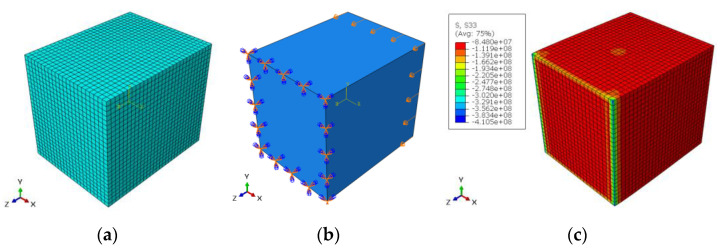
(**a**) FE model, (**b**) boundary conditions and (**c**) stress distribution for tangential direction.

For tangential direction, the dimensions of the selected sample in the three directions were: 8.9 × 9.83 × 11.45 mm when the experimental fracture stress was 96.01 MPa (see Table 3). The mesh model contained 15,950 elements when the element type was C3D20R (20-node quadratic brick). The number of nodes was: 69,692. The convergence study for element numbers gives less than 1% with element size = 0.0004 m. For the boundary conditions, the displacement in z-direction was: 0.000578 m in compression and the front surface in x–y plane was prevented from movement and rotation in x, y and z directions. Figure 9a–c show the FE model, the boundary conditions and the stress distribution for tangential direction, respectively.

### 4.4. Validation of the Proposed Formulation

In order to validate the resulting mathematical formulation (1), some samples were selected for the three directions and for the same density value. Table 1 shows the experimental values of failure stress for the three directions when considering the same density ($\rho =1{.8\;g/\mathrm{cm}}^{3}$) at the first displacement rate ($v_{1}=$ 0.5 mm/min). The failure stress value at the first displacement rate is considered to be the static stress $\sigma_{s}$.

Formulation (1) can be modeled to cover the three directions. Therefore, three mathematical formulations can be obtained. Next, some other samples were selected to check their position regarding the resulting curve. Table 4 shows the experimental, expected values using Equation (1) and numerical values using FEM of failure stress for the three directions considering the same density ($\rho =1{.8\;g/\mathrm{cm}}^{3}$) at other displacement rates ($v_{2}=$ 1 and $v_{3}=$ 2 mm/min). The result of the expected values was close to the experimental ones for the radial and tangential directions. In the radial direction, the experimental and numerical stresses have the closet values when comparing to the other directions, while in the axial direction the values are not close enough. However, it can be used as a suggested value in the design of prostheses processes, as it has been previously declared as one of the objectives of this work. Table 4 also shows variations between expected values obtained using Equation (1) and the FEM values, however, both values are close to the experimental results.

**Figure 10:**
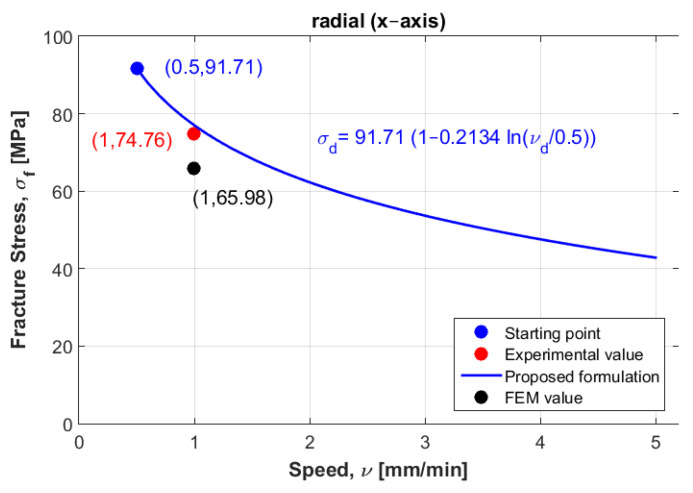
Curve modeling for the radial direction and the experimental point at the second displacement rate.

Figure 10 shows the curve modeling for the radial direction. It also shows the position of both experimental and numerical values at the second displacement rate ($v_{2}=$ 1 mm/min), when the experimental value of the failure stress was 74.76 MPa and numerical value was 65.98 MPa.

**Figure 11:**
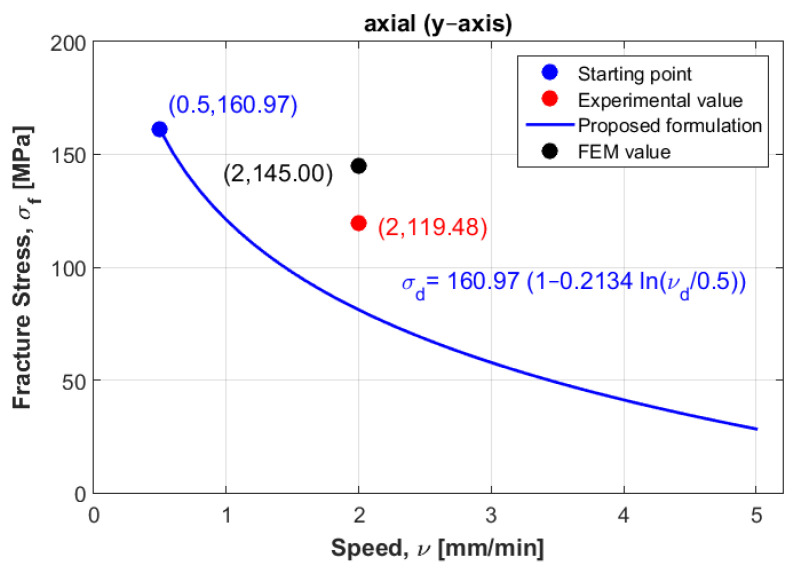
Curve modeling for the axial direction and the experimental point at the third displacement rate.

Figure 11 shows the curve modeling for the axial direction. It also shows the position of both experimental and numerical values at the third displacement rate ($v_{3}=$ 2 mm/min) when the experimental value of the failure stress was 119.48 MPa and numerical value was 145 MPa.

**Figure 12:**
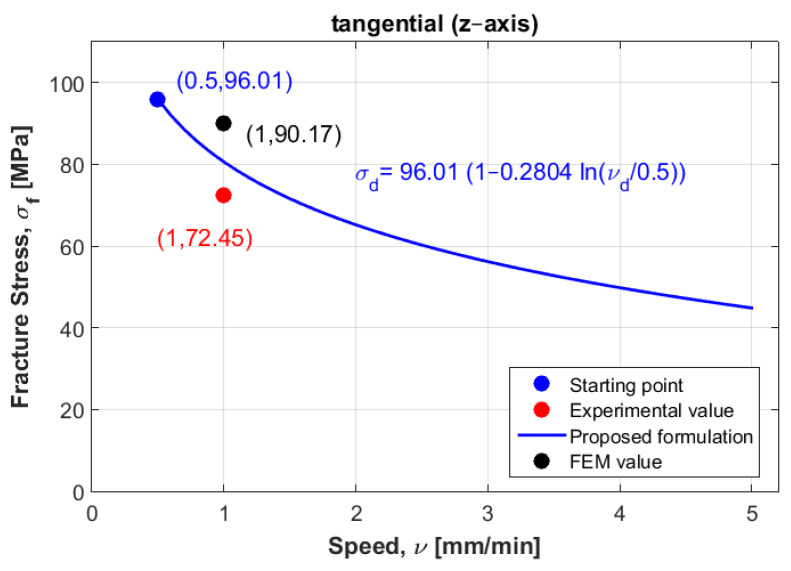
Curve modeling for the tangential direction and the experimental point at the second displacement rate.

Figure 12 shows the curve modeling for the tangential direction. It also shows the position of both experimental and numerical values at the second displacement rate ($v_{2}=$ 1 mm/min), when the experimental value of the failure stress was 72.45 MPa and numerical value was 90.17 MPa.

The expected values of failure stress obtained using formulation (1) were found to be in good agreement with the experimental values of failure stress, and were not far away from the numerical values in both radial and tangential directions (see Table 4 and Figure 10 and Figure 12), which might be due to the selection of the other samples at the same speed ($v_{2}=$ 1 mm/min) that were used in the mathematical model elaboration. However, for the axial direction, the numerical failure stress was found to not be close to the failure stress obtained from the experimental, which might be due to the sample being selected at a different speed ($v_{3}=$ 2 mm/min). Another reason this can be mentioned is that according to the finding of Nobakhti et al. [8], the mechanical properties change along the femur. The sample may have been taken from a different region. To solve this problem, more samples at different speeds and different regions were needed to reformulate the mathematical model. However, the authors believe that the suggested model can be used when performing reliability and optimization studies in prosthesis design.

## 5. Discussion

It is well known that the bone material structure is complex and shows an anisotropic mechanical response. In this work and for simplicity, the bone was considered to be homogeneous, and linearly elastic but not isotropic. Therefore, three directions were considered: radial, axial and tangential directions. The different curves of stress-strain relationships showed that the studied cortical bone was a brittle material, as no plastic strain was observed.

According to the old studies of Currey [18] and Wright and Hayes [19] on the bovine femur and of Crowninshield and Pope [20] on the bovine tibia, the ultimate stress increases when the strain rate increases. However, in recent studies of Brown at al. [16], it is mentioned that the ultimate stress values decrease in transversal and longitudinal directions as the strain rates increase. In this work, when carrying out several experiments on bovine femur and tibia at four speeds, the same findings were obtained in certain areas where the failure stresses decreased, while they increased in the others in the different directions (radial, axial and tangential). These results can be supported by the work of Ferreira et al. [5], who provided an interval between the maximum and minimum values for ultimate strength, strain at fracture in axial and transversal directions when their results were not statistically significant. When the characterization is carried out at the micro-structural level, Li et al. [7] concluded that the cortical bone mechanical properties vary not only from bone to bone, but in the same bone.

Therefore, we defined these mechanical properties at the macro-structural level, and the mathematical model was herein constructed according to the worst results, in order to obtain a reliable mathematical description. If the femur or tibia are divided into right and left sides, different behaviors can be found. The output of this study will be used in the prosthesis design. In this case, when implanting a prosthesis, the worst case must be considered to reduce the likelihood of failure. Therefore, some samples in three directions at three speeds were selected, considering the same density in each direction. To define the mathematical model, several trials were first carried out to find the best mathematical model fitting the selected points (samples). For example, when using the parabolic description, the error was largely reduced, and the curve passed through these three points. It was not a rational selection because the behavior of the failure stress may decrease and increase (up and down) in certain parts of the resulting curve. It was found that the fitting technique was not suitable to describe the model. In addition, according to Hansen et al. [9], it is shown that there is a strong effect of the strain rates when considering the post yield properties comparing to pre yield deformations. To formulate the mathematical model (1), all these considerations were taken into account when selecting the samples in Table 1.

Next, an approximation technique was used for all directions, whereby the failure stress values homogenously decreased when increasing the displacement rate values (see Figure 3). To validate these mathematical models, other samples were selected considering the same density for all directions to compare between their behaviors in dynamic cases. At the first speed, the static failure stress was considered as a starting point to model the different curves. Then, other samples with same density at different speeds were selected to test the error, these samples were not found far from the different curves, especially in the radial and tangential directions. In addition, a numerical study was carried out to compare to the experimental results. In order to perform this, calculations of Young’s modulus were needed for the three direction. Six samples were used at the same density to compute the mean values. According to the resulting Young’s modulus in the radial and tangential direction, the transversal isotropy was confirmed, since their values were almost similar. The same finding exists in literature when testing the human femur [21]. One of the limitations of is that the used samples (almost 100) had different density values, which can be an obstacle to perform a realistic statistical study by determining the data centrality (mean, mode and median) and variation (range, variance and standard-deviation). In order to perform this realistic study, we needed to obtain many bovine samples (same sex and age). To estimate the effect of the randomness of experimental results, an uncertainty analysis was needed [22]. A sensitivity analysis should be established in order to determine the most critical parameters regarding the experimental results [23,24]. However, this kind of study can be carried out using a big number of samples for the same density. In future work, the effect of uncertainty measurements can be carried out at each speed, in order to obtain reliable mathematical formulations.

One important finding is that the curves in the radial and tangential directions were parallel (see Figure 3), in which confirms that the transversal isotropy can be applied in dynamics as well as in statics. For Figure 10 and Figure 12, for the same density ($\rho =1{.8\;g/\mathrm{cm}}^{3}$), the difference between the curves in radial and tangential was small. When comparing FE results at the other speeds with the proposed curves and the experimental values in Figure 10 and Figure 12, the numerical values diverge largely when increasing the speed values, while the resulting curves were much closer to the experimental values. Since the worst scenarios of the experimental samples were taken into account, the reliability of the studied prosthesis can be increased when integrating the proposed formulation (1) into a reliability and/or optimization algorithms [3,4]. In references [3,4], the authors have formulated an empirical formula between Young’s modulus and failure stress, in order to integrate them into reliability and/or optimization algorithms. Here, the Young’s modulus was considered as a random variable, and any change in this variable will lead to a new constraint due to the relationship between the young modulus and the fracture stress. These developments were carried out considering static loading cases, which may lead to unrealistic results, since bone is almost always subjected to dynamic loads. Therefore, there was a need to develop new formulations that takes the dynamic effects into account. In this work, the developed formulation might be used as an indicator to predict fracture stress at different speeds, which enhances the opportunity to use these different speeds in the reliability and/or optimization algorithms. In addition, the transversal isotropy property is demonstrated here for dynamic loading cases as well as in static ones, which had been previously demonstrated [4].

Furthermore, the proposed formulations can be used to provide the expected stress value at the different speeds in the interval (0.5–5 mm/min), instead of preforming FE simulations. This may lead to some unreliable results, since the model of bone is very difficult in FE simulations, and many simplifications are required to do the FE simulations, such as ignoring the porosity. On the other hand, the proposed formulation is simple and can be at least used at the beginning of reliability and optimization algorithms as mentioned earlier.

There were some simplifications considered during this study, such as the density range tolerance and the limited number of samples. When selecting the samples, the objective was to establish a mathematical formulation for each direction at the same density ($\rho =1{.87\;g/\mathrm{cm}}^{3}$ for radial direction, $\rho =1{.70\;g/\mathrm{cm}}^{3}$ for axial direction, and $\rho =1{.69\;g/\mathrm{cm}}^{3}$ for tangential direction). Therefore, the density range tolerance was small (±0.05 g/cm^3^). However, when validating the obtained formulation, it was needed to select samples at the same density ($\rho =1{.8\;g/\mathrm{cm}}^{3}$) for the three directions, and also at several speeds. So, the density range tolerance was bigger (±0.5 g/cm^3^). To reduce the data variation, limited in this study to the measured samples, it would be necessary to test a larger number of samples to analyze the different directions and different speeds. It would be also important to cut samples from the same regions of the bone, considering age and sex, and then to sort according to density ranges. This is an important experimental program, which is definitely challenging, and this could be contributing to the results obtained in this preliminary work.

## 6. Conclusions

In this work, three curves describing the long bone behavior properties under different speeds at radial, axial and tangential directions are presented. These curves provide the best approximation and produce new formulations that relates the failure stress value in dynamics with failure stress in statics. The limitations caused by the available samples (sample density spread and speeds) can be overcome with a large experimental program, but also with a reliable FE model, tested with key measurements to reduce the needed ranges of the measured data. In this way, a robust correlation between fracture stress and Young’s modulus would provide a better relationship.

The behavior of the bone in the radial and tangential directions were almost similar, which gives an indication to consider the transversal isotropy in dynamics as well as in statics. This work paves the way for future developments, in which a statistical study is needed to provide more accurate formulations, describing the cortical and cancellous bone tissues. This can be effectively integrated into the different developments in the orthopedic prosthesis design, which is considered as a multidisciplinary strategy resembling several fields, such as medicine, mathematics, physics and chemistry.

## Figures and Tables

**Table 1:** Experimental and expected values using Equation (1) of the failure stresses for the three directions at three displacement rate values.

		Failure Stress (MPa)	
Direction	Speed (mm/min)	Experimental Values	Expected Values Using Equation (1)
Radial	0.5	107.80	107.80
	1	74.759	91.854
	5.0	59.980	54.830
Axial	0.5	146.55	146.55
	1	76.946	110.19
	5.0	35.790	25.779
Tangential	0.5	64.423	64.423
	1	32.779	51.902
	5.0	28.593	22.829

**Table 2:** Values of constant $C_{i}$ for the three directions radial, axial and tangential.

Direction	Radial	Axial	Tangential
$C_{i}$	−0.2314	−0.3579	−0.2804

**Table 3:** Experimental values of fracture stress at $\rho =1{.8\;g/\mathrm{cm}}^{3}$ and $v_{1}=$ 0.5 mm/min.

Direction	Radial	Axial	Tangential
Experimental Stress [MPa]	91.71	160.97	96.01
Numerical Stress [MPa]	82.14	188.4	84.8

**Table 4:** Experimental, expected value using Equation (1) and numerical values of fracture stress at $\rho =1{.8\;g/\mathrm{cm}}^{3}$ and $v_{2}=$ 1 and $v_{3}=$ 2 mm/min.

Direction	Radial	Axial	Tangential
Experimental Stress [MPa]	74.76	119.48	72.45
Expected Stress using Equation (1) [MPa]	77.00	81.104	80.611
Numerical Stress (FEM) [MPa]	65.98	145	90.17
Speed [mm/min]	1	2	1

## Data Availability

Not applicable.
